# The Fragility of Individual-Based Explanations of Social Hierarchies: A Test Using Animal Pecking Orders

**DOI:** 10.1371/journal.pone.0158900

**Published:** 2016-07-13

**Authors:** Ivan D. Chase, W. Brent Lindquist

**Affiliations:** 1 Department of Sociology, Stony Brook University, Stony Brook, New York, United States of America; 2 Graduate Program in Ecology and Evolution, Stony Brook University, Stony Brook, New York, United States of America; 3 Department of Mathematics & Statistics, Texas Tech University, Lubbock, Texas, United States of America; Cardiff University, UNITED KINGDOM

## Abstract

The standard approach in accounting for hierarchical differentiation in biology and the social sciences considers a hierarchy as a static distribution of individuals possessing differing amounts of some valued commodity, assumes that the hierarchy is generated by micro-level processes involving individuals, and attempts to reverse engineer the processes that produced the hierarchy. However, sufficient experimental and analytical results are available to evaluate this standard approach in the case of animal dominance hierarchies (pecking orders). Our evaluation using evidence from hierarchy formation in small groups of both hens and cichlid fish reveals significant deficiencies in the three tenets of the standard approach in accounting for the organization of dominance hierarchies. In consequence, we suggest that a new approach is needed to explain the organization of pecking orders and, very possibly, by implication, for other kinds of social hierarchies. We develop an example of such an approach that considers dominance hierarchies to be dynamic networks, uses dynamic sequences of interaction (dynamic network motifs) to explain the organization of dominance hierarchies, and derives these dynamic sequences directly from observation of hierarchy formation. We test this dynamical explanation using computer simulation and find a good fit with actual dynamics of hierarchy formation in small groups of hens. We hypothesize that the same dynamic sequences are used in small groups of many other animal species forming pecking orders, and we discuss the data required to evaluate our hypothesis. Finally, we briefly consider how our dynamic approach may be generalized to other kinds of social hierarchies using the example of the distribution of empty gastropod (snail) shells occupied in populations of hermit crabs.

## Introduction

The hierarchical differentiation of individuals within groups of humans and animals is a near universal phenomenon. Some individuals have greater, and others lesser, access to high quality material resources, status, or control over other group members [1–5]. The success of individuals within a hierarchical system has consequences for their opportunities to mate, their health, and their general life chances. Consequently, the study of hierarchical differentiation is a core area of interest in both the social and biological sciences.

### The Standard Approach

Broadly speaking, most social scientists and biologists use a standard approach in attempting to account for patterns of social differentiation. We make the following observations on this approach.

1. The approach conceives of a hierarchical pattern as a *static distribution* of individuals with varying amounts of something of value. For example, researchers represent income inequality by statistical distributions showing the percentages of individuals in a population with varying amounts of money at some particular time [1].
2. The approach assumes that micro-level processes involving individuals generate major features of the hierarchical patterns. To continue with the example of income inequality, econo-physicists propose that “random” economic transactions between individuals generate income distributions [1, 6]. Human capital theorists suggest that individuals having larger amounts of human capital—their education, training, health, etc.–to invest in the job market derive proportionally larger financial returns than those with smaller amounts of human capital [7, 8]. These theorists suggest that this differential return on investment generates the positive statistical skew of personal income and wealth observed in these distributions.
3. Researchers using this approach take a specific hierarchical pattern as given and attempt to reverse engineer the qualities or actions of individuals that produced the observed pattern. This is in contrast to attempting to infer the processes that might explain the pattern from direct observation of the hierarchical system itself. For example, instead of deriving its explanations from close observational studies of the careers of individuals over time, human capital theory simply assumes that the distribution of income derives from the investment of individuals in their human capital [7, 8].

In this paper we use the example of dominance hierarchies (pecking orders) in animals to evaluate the standard approach to hierarchical systems. Dominance hierarchies are a particularly good system for making this evaluation since they can be studied under carefully controlled laboratory conditions without the confounding condition of the subjects’ awareness of participating in an experiment that is often the case with humans. We use both experimental and analytical work from small groups of cichlid fish and hens in evaluating various explanations for the organization of dominance hierarchies based upon the standard approach (see references below). Our evaluation suggests that the standard approach does not work very well in accounting for the production of dominance hierarchies in these animals. Consequently, we propose a dynamics-based framework whose features contrast with each of the observations noted above for the standard approach. The new framework (1) considers a dominance pattern to be continuously dynamic rather than becoming static, (2) uses dynamic sequences of interactions rather than individual factors to account for the dynamic pattern, and (3) derives the dynamic sequences of interactions from direct observation of hierarchies as they form, rather than attempting to account for the pattern of a hierarchy after it has developed. We test the effectiveness of this new approach using a computer simulation, and we find a good fit between the simulation and the actual dynamics of dominance hierarchy formation in small groups of hens. We hypothesize that the rules of hierarchy formation described in the computer simulation are also used in small groups in many species of animals establishing hierarchies. Finally, we suggest that dynamics-based frameworks might better account for the organization of other kinds of social hierarchies, and we briefly describe how such a framework might be used in the investigation of the distribution of empty gastropod (snail) shell in groups of hermit crabs.

### The Standard Approach Applied to Animal Dominance Hierarchies

In this section we evaluate the three main features of the standard approach to hierarchical differentiation as traditionally applied to animal dominance hierarchies.

### Viewing Animal Dominance Hierarchies as Static, Individual-Based Distributions

We first show how researchers derive the usual static, individual-based view of dominance hierarchies from the underlying raw data of aggressive interactions. We then indicate the distortions resulting from this way of viewing the data.

**Fig 1:**
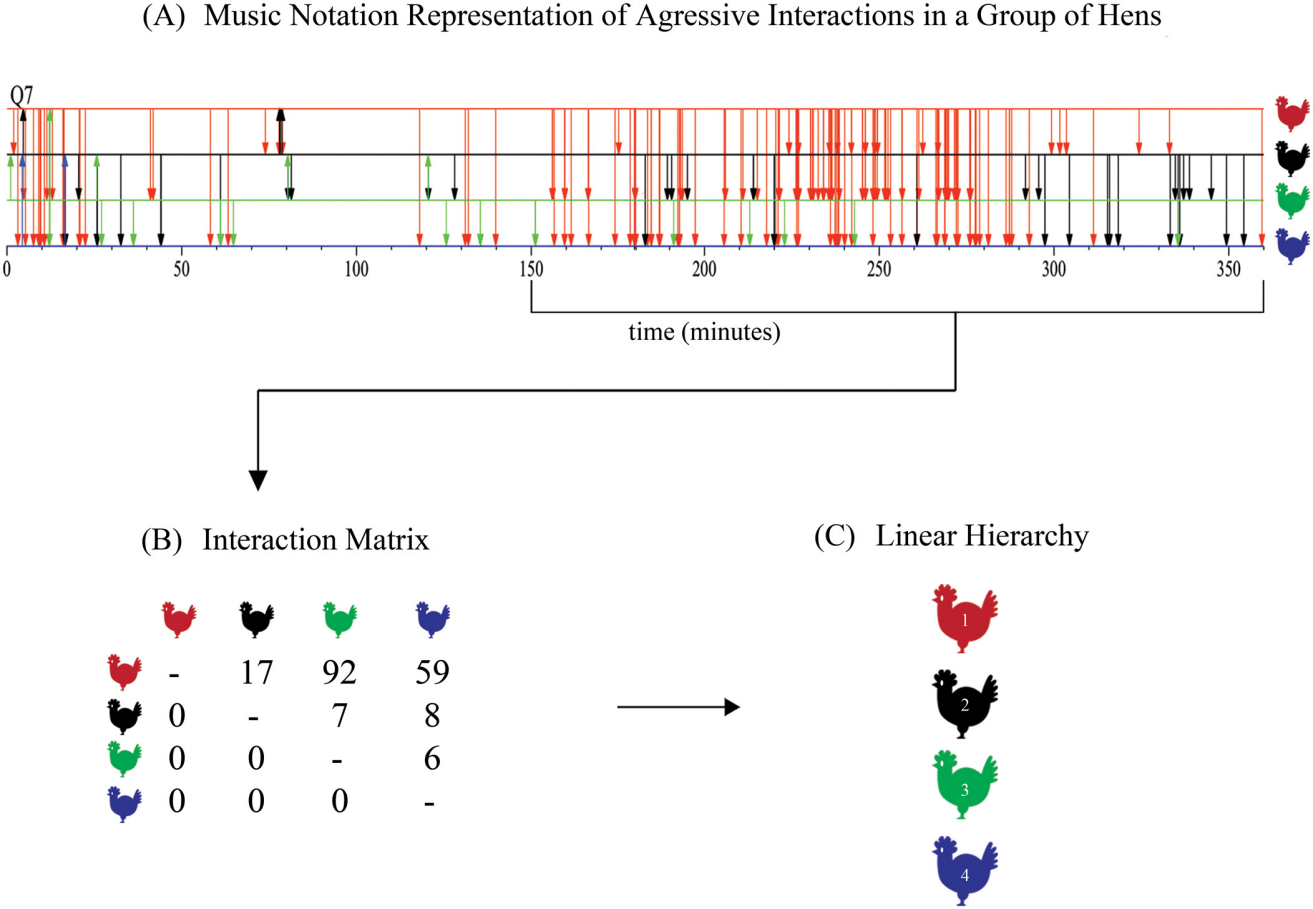
The steps required to derive a linear hierarchy from the raw data of aggressive interactions in a group of hens. (A) Music notation description of the observed aggressive interactions for the 6 hours of observation of hen group 7 on day 1. The hens appear as horizontal lines, the higher the line, the higher the rank of the hen during the period in which the hierarchy is stable (see text). Vertical arrows indicate an observed aggressive encounter at that time. The arrows are drawn from aggressor to recipient. When an aggressive act from a higher-ranking to a lower-ranking hen is followed closely in time by an aggressive act from the lower-ranking hen to the higher-ranking one, or vice versa, the two separate arrows indicating the acts may appear as a single double-headed arrow due to the restricted space allotted to the graph. (B) Frequencies of attacks during the observation of hen group 7 from 150 to 330 minutes. The numbers in the cells of the table indicate the frequency with which row hen i pecks column hen j. (C) The linear hierarchy suggested by the data from Fig 1(B).

Fig 1(A) uses “music notation” [9] to display a typical record of all aggressive interactions involving physical contact (chiefly pecks) among a group of four hens from their introduction over a six-hour period (see [10] for experimental details). The figure displays an initial, roughly 20-minute period during which the hens trade attacks. Subsequently, the counterattacks cease, then return again between the 80- and 120-minute marks, and then vanish once more. On the second day of observation (not shown) this group displayed two more periods of counterattacks. Following the standard procedure used in dominance hierarchy research, we abstract static “dominance relationships” from the dynamical raw data by summarizing the frequency and direction of attacks between the pairs of hens in a window of time with no or only a few counterattacks (e.g. the 3- hour period from 150 minutes through 330 minutes on day 1). The results are shown in Fig 1(B). If one hen in a pair does all or most of the attacks, we say that she “dominates” the other. We abstract once again to arrange the individuals by the number of other hens that they dominate to give the “dominance hierarchy” in Fig 1(C). This particular hierarchy is termed “linear”, since the individuals can be ordered from top to bottom by the number of other hens that they dominate. Researchers using these procedures often report linear hierarchies in small groups of animals—under some eight or ten members—for a broad range of species including some insects and reptiles, and many fish, birds, and mammals, including human children ([2] and references cited therein). The hierarchies in some species such as elephants, hyenas, and dolphins are organized by both dominance relationships and alliances and are too complex to be characterized as simply linear [11–13] Our discussion in this paper does not apply to such hierarchies.

Two important consequences arise from using these standard procedures. First, the procedures promote the view that aggression in groups reaches a stable, static pattern [14, 15], particularly a linear hierarchy, and rarely departs from it. Consequently, virtually all attempts to account for the organization of hierarchies have concentrated upon linear structures (for an exception see [16]). However, aggressive interactions in groups continue, even after a linear or other structure has been identified using the standard procedures. Through these interactions individuals can reverse the direction of their relationships with other individuals, and structures can go from linear to non-linear. In a linear hierarchy, all subgroups of three animals (triads) have a transitive relationship (i.e. A ⇒ B, B ⇒ C, A ⇒ C). If a triad is not transitive then it is intransitive (i.e. A ⇒ B, B ⇒ C, C ⇒ A). A non-linear hierarchy has at least one triad with an intransitive relationship.

For example, in the group shown in Fig 1, the hierarchy switches between linear and non-linear five times on the first day of observation and four times on the second day of observation. In the 14 groups of hens from which the interaction record in Fig 1(A) was drawn, about half of the groups developed non-linear hierarchies at least once during the 12 hours they were observed [17], and other, longer-term studies, indicate that changes in hierarchy structures are common [18, 19]. To use the standard procedures is to ignore the dynamic, changing nature of aggressive interaction in groups.

The second consequence is that the standard procedures promote the use of individual-centered explanations for the organization of aggressive behavior in groups. That is, if hierarchies are seen as distributions of individuals with varying amounts of dominance, it seems natural to account for these varying amounts by differences among the individuals—in their attributes, actions, decisions, etc. While it seems reasonable, these attempts only work well in limited circumstances.

### Using Individual-Based Explanations of Hierarchy Structures

Explanations of hierarchy structures based upon individuals fall into two broad types. The first type, referred to as the “prior attributes hypothesis,” proposes that the differences in dominance ability that individuals possess *before* introduction predetermine their ranking in a linear hierarchy [2]; i.e. the individual highest in dominance ability takes the top position in the hierarchy, the individual next highest in ability takes the second position, and so on. Under this explanation, a linear hierarchy is simply a reflection of a prior linear ranking of the individuals based upon their differences in experiential, genotypic, and phenotypic attributes.

The second type of explanation based upon individuals, the individual action hypothesis, proposes that the behaviors and decisions that the individuals undertake *during* group formation explain linear hierarchies [10, 20]. In this category of explanation, researchers have examined winner-loser models most intensively. In these models individuals achieve their ranking as consequences of winning or losing pair-wise encounters during hierarchy formation; e.g., [21–25]. Specifically, an individual winning one encounter increases its probability of winning its next contest, and an individual losing an encounter decreases its likelihood of winning a subsequent contest. Some models in this category incorporate bystander effects—individuals adjust their behavior toward other individuals based upon observation of the outcomes of the contests involving those other individuals [26]. Still other models use game theory to prescribe how individuals might interact to generate linear hierarchies [27].

Experimental work indicates that both the prior attributes hypothesis and winner-loser models work extremely well in isolated pairs of individuals but not in small groups in some species. For example, in one experiment isolated pairs of cichlid fish met to form a dominance relationship [28]. They were then separated for two weeks—a period of time sufficient for them to forget one another as individuals [29, 30]. The same pairs were then reassembled to form a second relationship. The researchers found that the fish that dominated in the first meeting also dominated in the second meeting in 94% of the pairs. Differences in attributes appeared to be stable over the two-week break and appeared to determine which individual would be dominant in a pair.

The researchers then put groups of four fish through the same procedure and looked at dominance between each of the pairs making up a group [28]. For example, if fish A dominated fish B in the first assemblage of the group A, B, C, D, did she also dominate B in the second assemblage of the same four fish (and so on for all the possible pairs in the group)? In the groups of four, the same fish dominated in both meetings in only 75% of the pairs, significantly different, from the rate observed in isolated pairs. In addition, 73% of the groups formed different hierarchies the second time they met with two, three, or all four fish having different ranks [2, 28].

In an experiment examining the loser effect in isolated pairs in a species of cichlid fish (this species did not show a winner effect in isolated pairs or groups), prior losers lost 87% of the time in isolated pairs. However, prior losers only lost in 59% (not significantly different from the 50% expected due to “pure chance”) of their contests in groups of four fish [28]. In summary, experiments show that differences in attributes and in prior aggressive experience are highly predictive of dominance outcomes for individuals in isolated pairs but not for individuals in groups as small as four members.

Analytical work also demonstrates the inadequacy of both the prior attributes hypothesis and winner-loser models for explaining linear hierarchies in small groups of animals. Mathematical and statistical work [31, 32] shows that accounting for even almost-linear hierarchies by differences in prior attributes requires stringent mathematical conditions, and experimental data indicates that these conditions are rarely fulfilled. This problem is especially acute in larger groups [33]. Similarly, analytical work [34] demonstrates that the main mathematical components of three prominent winner-loser models of hierarchy formation are poor fits to the measurement of those components in groups of four hens forming hierarchies.

### Inferring Mechanisms as an Inverse Problem rather than from Direct Observation of Group Interaction

Taking a particular outcome, such as a linear hierarchy, as given and attempting to reverse engineer what processes might produce such a distribution is known more formally as a an inverse problem approach. In general, solving inverse problems correctly can be difficult; no less so in the case of the organization of aggressive behavior in groups. Such a solution is often non-unique—more than one set of micro-level processes can generate the same group-level pattern. Those processes can be of very different sorts as indicated by the prior attributes and winner-loser models of hierarchy formation. Even within a specific model, different sets of parameter values can produce the same group-level pattern. And, most important of all, without experimental validation that a particular set of processes actually matches other observable features (besides the final outcome state) of the formation of a group-level pattern, it is difficult to know whether the proposed processes are actually used.

## A Dynamical Model of the Organization of Aggressive Behavior in Groups

We introduce a new, dynamical model of the organization of aggressive behavior in animal groups. First, rather than seeing a static, linear hierarchy as the pattern to be explained, the dynamical model views the hierarchy as a dynamic network of aggressive interactions among group members that is updated with each successive act. The hierarchy of this network can stay the same over a period of time or it can change, sometimes radically. Second, the new model does not rely upon individuals as units of explanation. Rather, the model explains the dynamic structure of the network by dynamic processes of interaction occurring in subgroups of two and three animals. In this way the model is related to the use of static network motifs, patterns of interconnection among network nodes occurring at significantly high rates, to explain static network structures (e.g. see [35] and [36] for general references; [16] for a specific application to dominance hierarchies), but here we use what could be called dynamic network motifs (transitive and intransitive triads—see following) to account for dynamic network structures. Third, the dynamical model avoids the inverse tactics of the various individual-based explanations by directly inferring the rules for updating interaction networks from data on animals as they form groups. These rules are derived from a series of studies on the dynamics of aggressive interactions in chickens [17, 34].

More generally, our model falls within the tradition of social scientists exploring the connection between smaller-scale social phenomena involving, for example, individuals, subgroups, and families, and larger-scale phenomena such as larger groups, political movements, and companies. This tradition is often referred to as the investigation of the “micro-macro connection”. A particularly relevant example of research within this framework is Johnsen’s [37] work on explaining the static structures of friendship ties in larger human groups from analytically, not empirically derived small-scale processes in the component triads composing the groups. Martin [38] provides an extensive theoretical and empirical discussion of the connection between patterns of interaction and social structures with many human and some animal examples.

### Rules of Interaction

We hypothesize that exactly two rules of interaction are necessary and sufficient to generate the dynamic networks of aggressive interaction found in many species of animals forming small groups.

#### Rule 1

There are aggressive acts between every pair of animals comprising a group.

This rule, that all individuals in a group interact, enables a description of animal relationships via tournament graphs [39]. In a tournament graph there is a directed edge between all pairs of vertices. A vertex represents an individual animal, and a directed edge represents the most recent recorded attack between two animals (denoted as A ⇒ B). A linear hierarchy corresponds to a transitive tournament graph—a graph in which all subgroups of three vertices (triads) have a transitive relationship (i.e. A ⇒ B, B ⇒ C, A ⇒ C). As indicated above, if a triad is not transitive then it is intransitive (i.e. A ⇒ B, B ⇒ C, C ⇒ A). All triads in a linear hierarchy have transitive configurations. A hierarchy is non-linear if it contains at least one intransitive triad.

#### Rule 2

The rate at which intransitive configurations of attacks are converted to transitive ones is much higher than the reverse—the rate at which transitive configurations of attacks are converted to intransitive ones.

Rule 2 assures that any non-linear hierarchies will quickly revert to linear ones and that, at most points-in-time, hierarchies will have linear structures.

It should be noted that we are now using the terms “linear hierarchy” and “non-linear hierarchy” in a much more dynamic sense. Rather than a linear hierarchy referring to a static structure derived from a summary of interactions over some reasonably long window of time, we now use the term to indicate the current hierarchical organization resulting with each new aggressive encounter in a group. For example, suppose that the most recent attacks among group members were: A ⇒ B, A ⇒ C, A ⇒ D, B ⇒ C, B ⇒ D, and C ⇒ D. This four-animal hierarchy is linear. Assume next either:

A ⇒ B—Under our dynamic view, the hierarchy remains linear as this attack merely repeats an established direction of attack in an already linear hierarchy; or

C ⇒ A—The ABC triad would now be intransitive and the overall group hierarchy structure would be non-linear.

### Markov Chain Computer Simulations

Using computer simulations on tournament graphs we demonstrate that these two rules are sufficient to generate dynamical records of interaction which, when abstracted, give hierarchies that are mostly linear but are sometimes briefly non-linear, as in actual small groups. Each computer simulation began by forming a random network of attacks among all pairs composing a group of *n* animals in accordance with Rule 1. Thus a simulation started by choosing, with equal likelihood, an initial random tournament graph from all possible tournament graphs on *n* vertices. The probability of starting with a linear hierarchy is given by

$$P\left(\text{linear}\right)=\frac{n!}{2^{\left(\begin{array}{c} n\\ 2 \end{array}\right)}}=\frac{n!}{2^{n(n-1)/2}}.$$ (1)

The numerator is the total number of possible linear tournament graphs on *n* vertices; the denominator is the total number of possible tournament graphs on *n* vertices. Evaluation of Eq (1) indicates that most simulations involving 4 or more animals began with a network in which one or more triads have an intransitive configuration of attacks.

In groups of animals, processes of interaction encourage the initial formation of more transitive attack configurations than would be achieved if attacks were random [34]. Consequently, the network of attacks in our simulation usually started with more intransitive configurations than would be observed in real groups. We began our simulations this way in order to demonstrate the efficacy of Rule 2 in generating linear hierarchy structures, despite the handicap of “excess” initial intransitive configurations.

Let *α* = *P*(*I* → *T*) denote the probability for converting an intransitive triad to a transitive one and *β* = *P*(*T* → *I*) be the probability for converting a transitive triad to an intransitive one. After choosing the initial network, the simulation used a Markov process for Rule 2 to explore how a specific choice for the values *α* and *β* affected the linearity of the resulting hierarchy structures. (The Markov process ensured, at each step, that Rule 1 always held.) More specifically, the simulation took the initial network of attacks, selected a triad at random (equi-likelihood), picked a random number *y* (between 0 and 1 using a flat random number generator), and checked whether the triad was transitive or intransitive. If the triad was transitive, and if *y* ≤ *β*, it converted the triad to an intransitive one through a counterattack between a specific pair of vertices. (For example, in the transitive triad *A* ⇒ *B*, *B* ⇒ *C*, *A* ⇒ *C*, only the counter attack *C* ⇒ *A* will make the triad intransitive.) If *y* > *β*, the simulation did not change the triad.

If, however, the randomly chosen triad was intransitive, and if *y* ≤ *α*, the simulation converted the triad to a transitive one by changing, at random, one of the three attack directions. If *y* > *α*, the simulation did nothing to the triad.

The simulation continued, at each step choosing a triad at random, determining whether it was transitive or intransitive, and deciding whether to convert the triad depending upon a new randomly chosen value for *y* and the pre-selected target conversion probabilities *α* and *β*. Each simulation continued in this manner for 1,000 *τ* steps (where *τ* was the number of triads in the network). After each step the simulation examined the effect of any action on the triad under consideration by noting whether the overall hierarchy structure became/remained linear or non-linear. We ran many simulations with the same choice of *n*, *α* and *β*, starting each simulation with a new, randomly generated network to ensure an adequate sampling of initial network conditions. Overall, for each choice of *n*, *α* and *β*, we performed 1,000 *τ* simulations, calculating the fraction of steps in all the simulations in which the hierarchy was linear. Thus each calculation in our results below is based upon 10^6^*τ*^2^ observations (1,000 *τ* simulations times 1,000 *τ* steps for each simulation) of evolving interaction records.

## Results

### Analytical Results for Groups of Size 3

Table 1 displays the outcome of the simulations for *n* = 3, *τ* = 1 (3 animals forming a single triad). The analytical results are known for this Markov process when the number of observations is very large. If we define *P*(*T*) as the probability of observing a linear hierarchy over the course of the set of simulations, then

$$P\left(T\right)\approx \frac{\text{number of observations after which the hierarchy is linear}}{10^{6}\tau^{2}}.$$ (2)

For *n* = 3 the analytic solution is

$$P\left(T\right)=\;\frac{\alpha }{\alpha +\beta },\;\;\;\text{for}\;\alpha >0,\;\beta >0$$ (3)

If *α* = *β* = 0 then the analytic value for *P*(*T*) is given by the appropriate value from Eq (1). Comparison of Eq (3) with the results in Table 1 gives an estimate of the finite-sampling error, which is very low. The largest absolute-value difference between the analytic answer Eq (3) and the results in Table 1 is only 0.75%, which occurs for the entry *α* = 0, *β* = 0.1. This computation, with no direct probability for converting an intransitive triad to transitive requires many more than 1,000 *τ* steps per simulation to achieve an adequate sampling and come into agreement with Eq (3). (When the calculation for Table 1 was redone with the number of simulation steps increased to 10,000 *τ*, the largest absolute-value difference was observed to drop to 0.09%.)

**Table 1:** Observed P(T) for Three Animals.

*T* → *I* transition probability *β*	*I* → *T* transition probability *α*				
	0.0	0.1	0.5	0.9	1.0
**0.0**	74	99.75	99.95	99.97	99.98
**0.1**	0.7561	50.35	83.35	89.97	90.92
**0.5**	0.1509	16.66	50.02	64.29	66.68
**0.9**	0.0826	10.08	35.74	49.99	52.63
**1.0**	0.0756	9.181	33.36	47.37	50.00

The observed P(T) in percent for a Markov chain simulation for a single triad of three animals.

### Simulation Results for Groups of Size 3–7

**Fig 2:**
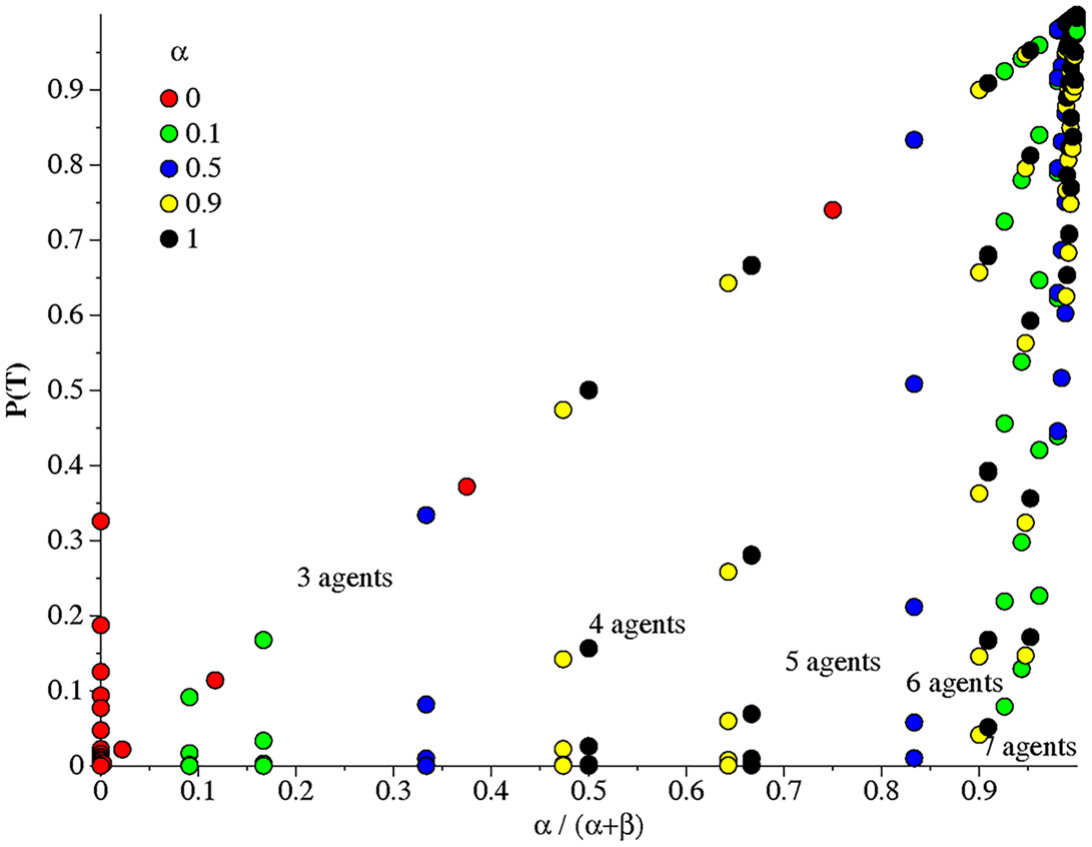
Probability of having a linear hierarchy. The probability P(T) of having a linear hierarchy (transitive network) is shown as a function of the ratio *α*/(*α* + *β*) of the transition probabilities *α* = *P*(*I* → *T*), *β* = *P*(*T* → *I*) for individual triads.

Fig 2 summarizes the results of the computations using groups of 3 through 7 animals for values of 0.0 ≤ *α* ≤ 1.0 and 0.0 ≤ *β* ≤ 1.0. The figure plots *P*(*T*) versus the ratio *α*/(*α* + *β*) for different values of *α*. Each point on the graph represents a computation for a fixed number of animals and fixed values of *α* and *β*. The results show that, for a given number, *n*, of animals, *P*(*T*) only depends on the ratio *α*/(*α* + *β*) and not on the separate values of *α* and *β*, i.e.

$$P\left(T\right)=f_{n}\left(\frac{\alpha }{\alpha +\beta }\right).$$ (4)

Unlike the case for *n* = 3, for *n* > 3 the function *f*_*n*_ is stronger than a single power law and surely depends on the value for *n*. We do not yet know the functional form for *f*_*n*_.

Fig 2 clearly shows that *P*(*T*) → 1 is only achieved if *α*/(*α* + *β*) → 1; i.e. if Rule 2, *β* ≪ *α*, holds. As *n* increases this requirement becomes more stringent; that is, for a fixed value of *α*, as *n* increases *β* must decrease rapidly to maintain a constant value of *P*(*T*). For example, in groups of five, even if intransitive to transitive conversion is a high value of *α* = 0.95, a probability of *β* = 0.008 results in hierarchies being linear only 90.5% of the time while for the same value of *α*, in groups of seven, *β* must fall to a value of 0.002 to achieve linear hierarchy 90.9% of the time.

In some sense the results in Fig 2 are intuitive and not surprising. Linear structures by mathematical definition have no intransitive triads, only transitive ones, and operations that remove intransitive triads propel hierarchies toward linearity. *But the important point here is that the simulations demonstrate that two rules for the dynamics of interaction can*, *on their own*, *produce linear structures without any need for models that rely upon the attributes*, *actions*, *or decisions of specific individuals as agents*.

### Empirical Evidence that Animals Follow the Two Rules

#### Rule 1

As specified in Rule 1, reports in the animal behavior literature indicate that tournament hierarchy structures—dominance relationships present between all pairs of individuals in the group—occur in small groups of under some 8 or 10 members in a wide variety of species [2, 40]. However, in groups larger than this, researchers often do not observe interactions among some pairs [41].

#### Rule 2

As far as we are aware, only one study [10] has collected interaction data during hierarchy formation that can be analyzed to evaluate Rule 2. An analysis of these data [17] found that 15 triads (27%) of the total of 56 triads in the 14 groups of hens did develop intransitive triads at some point during the 2 days of observation on each group. All but one of these triads later converted to transitive triads; the one that did not convert occurred shortly before the end of the observation period on that group of four hens. The analysis [17] discovered that these intransitive triads tended to last only a short time. On average only 5.2 interactions occurred in a group before an intransitive configuration was converted into a transitive one. By comparison transitive configurations were much more stable. They lasted on average for 54.3 interactions– 10 times longer—before being converted into a different transitive configuration (e.g. from *A* ⇒ *B*, *B* ⇒ *C*, *A* ⇒ *C* to *A* ⇒ *B*, *C* ⇒ *B*, *A* ⇒ *C*) or into an intransitive one. The median time before conversion of an intransitive triad was 2 min 4 sec while for a transitive one it was 49 min 22 sec—a factor of 23.9 times longer.

## Discussion

In this paper we have used the organization of aggressive behavior in animal groups as a test case to evaluate the standard approach employed by many researchers in biology and the social sciences for explaining patterns of social hierarchy. This standard approach is distinguished by three tenets: visualizing an inequality pattern as a static distribution of individuals with varying amounts of something of value, assuming that the qualities or actions of individuals explain the form of the distribution, and using inverse methods rather than direct observation to develop explanatory models.

We used both experimental and analytical research to evaluate the prior attribute hypothesis and winner-loser models. These two frameworks follow the three tenets of the standard approach and are the most prominent models for explaining the forms of dominance hierarchies. Our evaluation revealed that while these two frameworks predict dominance outcomes in isolated pairs of animals, they did not successfully account for the organization of dominance behavior in groups. Given the lack of support seen in the models for the three tenets of the standard approach, we proposed a new approach that viewed the organization of dominance behavior in small groups of animals as a dynamic network of aggressive interactions that can, and do, change their structure over time. Motivated by studies [10, 20, 34, 42] of the dynamics of hierarchy formation in small groups of chickens, we have suggested that two simple rules for the dynamics of interaction generate the changing structural forms of these dynamic networks. We used these rules in Markov chain computer simulations to demonstrate that they were sufficient to produce *dynamic* hierarchy structures (now defined for each occurrence of an aggressive interaction in a group rather than over a longer window of time as in the standard approach) that were mostly linear, but occasionally non-linear, as in real groups. We found excellent empirical support for the widespread use of Rule 1 in small groups of animals across species and good tentative support for the occurrence of Rule 2.

We now raise two questions about the use of the individual-centered approach in dominance behavior, apply the dynamical model to make three predictions, describe the data needed to evaluate our model in small groups of other species forming hierarchies, and finally, briefly discuss the generalization of our dynamics-based approach to other kinds of social hierarchies using the example of the distribution of empty gastropod shells in hermit crabs.

### Questions about the Use of the Individual-Centered Approach

#### Q1. Could an individual-based model account for the organization of aggressive behavior in groups of animals?

Given that the organization of aggressive behavior in groups results from the actions of individuals, it seems reasonable to attempt to describe this organization in terms of individual-based attributes, decisions, actions, etc. For example, Rule 1 could be restated as “Individuals do not limit with whom they interact” and Rule 2 as “Individuals have a strong tendency to take actions toward other individuals that produce transitivity in the majority of triads.” This, however, would not convert our dynamics-based approach into a true individual-based one since such an individual-centered model would have to successfully predict each interaction of each individual in a group over the course of an entire interaction record of the sort shown in Fig 1(A). Given the already demonstrated incapacity of individual-based models to make good predictions for the dominance outcomes of individuals in the much simpler static hierarchy structures of the standard approach, it is difficult to imagine an individual-based model being successful in making accurate predictions in an even more demanding situation.

In our view aggressive interactions in small groups constitute a complex system (see [43] for a similar claim that aggressive behavior in groups constitutes a complex system). Although complex systems are difficult to define precisely, one hallmark that has been suggested is that in these systems our usual notions of cause and effect break down [44]. The intricacies of the many interactions among the elements of these systems make it difficult to delineate clearly which particular actions of which particular elements of the system lead to specific outcomes. While, traditionally, complex systems are considered to be composed of many interacting agents, we propose that the convolutions of interaction, even among a relatively small number of animals, produce the same general quality of obscuring cause and effect. As in the study of other complex systems, our way to understand the production of system-level patterns has been to focus on rules governing the dynamics of interaction rather than on attempts at element-level explanations (see [45] for a related argument for not using individual organism models in ecology).

#### Q2. Are we suggesting that individual factors have no influence on dominance behavior?

No. We argue that the efficacy of individual-based approaches in understanding the organization of aggressive behavior in groups is necessarily limited. We do not claim that these individual-based approaches give us no information about dominance outcomes, rather we argue that they provide limited information. Differences in attributes and winner and loser effects are quite good at predicting the outcomes of contests within isolated pairs of animals, and as the results of [2, 28] indicate, differences in attributes can partially account for individuals’ ranks within hierarchies in small groups. Our point, stated in the previous paragraph, is that the jump from two individuals in a group to three or four is truly substantial. The high variability in individual outcomes associated, for example, with differences in attributes or in losing contests in groups (of more than two members) undermines the effectiveness of the individual-centered approaches. As is often the situation in the physical sciences, it really is the case that “more is different” when it comes to aggressive behavior in groups [46].

While the two rules for the dynamics of interaction that we have proposed are not direct expressions of selective behavior on the part of individuals, we assume that the rules emerge from adaptive behavior on the part of individuals. However, given the barriers that we have already mentioned to developing an adequate individual-based model for the organization of aggressive behavior in groups, we think that it would be very difficult to develop a model based directly upon the adaptive behavior of individuals that could accurately predict the successive, act-by-act information needed to account for the changing structure of dynamic networks in real groups of animals (see also [43]).

### Predictions from the Dynamic Model

We offer three predictions based upon our thesis that two rules for dynamic interaction generate behavioral records that can be abstracted to give dynamic networks that are mostly linear but occasionally non-linear. First, we conjecture that the reason that small groups across many species have the same pattern of organization in their aggressive behavior is because all these species use the same two rules of dynamic interaction described here. We are encouraged in this prediction by Shizuka & McDonald [16] who find strikingly similar network motifs in static dominance hierarchy structures across taxa, group size, and study settings (captive or wild groups). We hope that researchers will provide detailed data sets of the dynamics of interaction in small groups over extended periods of time in order to evaluate this conjecture.

Second, we expect that animals in larger groups will follow our two rules, but at lower levels than those in smaller groups. Specifically, we expect that not all pairs of animals will interact and that the ratio of the rate by which intransitive triads are converted to transitive ones compared to the rate by which transitive ones are converted to intransitive ones will be considerably lower in larger groups (see, for example the analysis in [47]). Analysis [41] of the static hierarchy structures in 40 published datasets provides tentative support for these predictions. They find no evidence of dominance relationships for some members of these groups and, while many of the groups’ hierarchy structures are not linear, they do contain significantly fewer intransitive triads than expected by chance (also see [48].

Third, while there is considerable evidence across a number of species that animals can infer transitivity from observing the interactions of other individuals (e.g. [49], [50], and [51]), there is, as far as we know, no experimental evidence that animals can infer *intransitivity* in relationships. However, the most direct way that Rule 2 could emerge from individual behavior would be for animals to infer intransitivity, as well as transitivity, from observing interactions. Consequently, we predict that animals in a variety of species can deduce this property of relationships.

### Application of the Dynamics-Based Model to Other Species of Animals Forming Hierarchies in Small Groups

We predicted above that many species of animals will form hierarchies in small groups following the two rules we have described here. In order to test this prediction researchers would have to collect data sets similar to the one in hens referred to here. In that data set the researchers: (1)observed hens that had not met previously, (2) recorded *all* instances of a set of aggressive acts deemed to be the most important in dominance interactions in chickens, (3) noted the time of occurrence of each aggressive act, and (4) logged the acts continuously, without breaks, during the course of an observation period (see [10] for more details). With a data set of this sort, a researcher can write a computer program to indicate when the initial attacks in a component triad first fill in and how long that configuration lasts until it is changed into a different triadic configuration (see [17] for further information).

### Generalization of the Dynamics-Based Approach to Other Kinds of Social Hierarchies

Given our conclusions relative to dominance behavior, we venture that many other forms of social hierarchy can be better conceived of as dynamic structures explained by small-scale dynamical processes. One such type of social hierarchy is the unequal distribution of material resources in populations of humans and animals. While it might be very difficult to carry out controlled experiments on the dynamics of resource distribution in large human inequality systems, it would be easier to investigate them in animals. One possible system is the occupation of empty gastropod (snail) shells in hermit crabs [52]. Unlike regular crabs, hermit crabs have soft abdomens, and as an adaptation, live in and carry around empty snail shells as portable shelters. Hermit crabs grow over the course of their lives and must periodically find and move into larger shells. Most species of crabs cannot kill snails for their shells, and empty shells appear to be scarce for most populations of hermit crabs.

Both field and laboratory research already indicate that hermit crabs obtain new, vacant gastropod shells through a dynamical form of interaction and not just through individual-based processes [52]. This form of dynamical interaction is a vacancy chain [52–54]. In a vacancy chain a new, empty gastropod shell is taken by a first crab that leaves its old shell behind. The first’s old shell is taken by a second crab leaving its prior shell behind, and so on. Vacancy chains are also important for humans in the distribution of such resources as houses and apartments, cars, and certain kinds of jobs [55]. Intriguingly, preliminary research suggests that the average number of individuals getting new resource units in vacancy chains is approximately the same in human and hermit crab populations [55].

In order to track the importance of dynamical processes such as vacancy chains for the overall form of the distribution of sizes of snail shells occupied in populations of hermit crabs, researchers could set up groups of young hermit crabs occupying shells of uniform size. They could then introduce empty gastropod shells of set sizes periodically and determine the effect, if any, of vacancy chains, or other observed dynamical processes, upon the forms of the group-level distributions of shells occupied over time.

## Conclusions

Regardless of the future support for the specific conjectures raised in this paper, we concur with Blonder et al. [14], Pinter-Wollman et al. [15], and Shizuka & Mc Donald [16] that the time has come for a new approach to the study of social organization in human and animal groups. That approach needs to be rooted in the observation of the dynamics of real creatures forming social systems, and we need to base our explanations of the organization of these systems upon those dynamics.

## References

[1] Chakrabarti BK, Chakraborti A, Chakravarty SR, Chatterjee A. Econophysics of income and wealth distributions. Cambridge, U.K.: Cambridge University Press; 2013.

[2] Chase ID, Tovey C, Spangler-Martin D, Manfredonia W. Individual differences versus social dynamics in the formation of animal dominance hierarchies. Proc Nat Acad Sci. 2002;99(8):5744–9. 10.1073/pnas.082104199 11960030 PMC122842

[3] Tilly C. Durable inequality. Berkeley: University of California Press; 1998.

[4] Vance RR. Competition and mechanism of coexistence in three sympatric species of intertidal hermit crabs. Ecology. 1972;53:1062–74. 10.2307/1935418

[5] Gould RV. The origin of status hierarchies: A formal theory and empirical test. Am J Sociol. 2002;107(5):1143–78. 10.1086/341744

[6] Yakovenko VM. Applications of statistical mechanics to economics: Entropic origin of the probability distributions of money, income, and energy consumption In: Taylor L, Rezai A, Michl T, editors. Social fairness and economics: Economic essays in the spirit of Duncan Foley. Frontiers of Political Economy. New York: Routledge; 2013 p. 53–82.

[7] Becker GS. Human capital. Chicago, IL: University of Chicago Press; 1964.

[8] Mincer J. Investment in human capital and personal income distribution. J Polit Econ. 1958;66:281–302. 10.1086/258055

[9] Chase ID. Music notation: A new method for visualizing social interaction in animals and humans. Front Zool. 2006;3(18). 10.1186/1742-9994-3-18 PMC1665449 17112384

[10] Chase ID. Dynamics of hierarchy formation: the sequential development of dominance relationships. Behav. 1982b;80:218–40. 10.1163/156853982x00364

[11] Wittemyer G G WM. Vollrath F. Douglas-Hamilton I. Social dominance, seasonal movements, and spatial segregation in African elephants: a contribution to conservation behavior. Behav Ecol Sociobiol. 2007;61:1919–31. 10.1007/s00265-007-0432-0

[12] Frank LG, Holekamp KE, Smale L. Dominance, demography, and reproductive success of female spotted hyenas In: Sinclaire ARE, Arcese P, editors. Serengeti II: Dynamics, management, and conservation of an ecosystem. Chicago: University of Chicago Press; 1995 p. 364–84.

[13] Wiszniewski J, Brown C, Möller LM. Complex patterns of male alliance formation in a dolphin social network. J Mammal. 2012;93(1):239–50. 10.1644/10-MAMM-A-366.1.

[14] Blonder B, Wey TW, Dornhaus A, James R, Sih A. Temporal networks and network analysis. Methods Ecol Evol. 2012;32(221–233). 10.1111/j.2041-210X.2012.00236.x

[15] Pinter-Wollman N, Hobson EA, Smith JE, Edelman AJ, Shizuka D, de Silva S, et al The dynamics of animal social networks: Analytical, conceptual, and theoretical advances. Behav Ecol. 2014;25(2):242–55. 10.1093/beheco/art047

[16] Shizuka D, McDonald DB. The network motif structure of dominance hierarchies. J Roy Soc Interface. 2015;12:20150080 10.1098/rsif.2015.0080 25762649 PMC4387537

[17] Chase ID, Lindquist WB. Dominance hierarchies as a model of social structure in small groups In: Hedstrom P, Bearman P, editors. The Oxford handbook of analytical sociology. Oxford, UK: Oxford University Press; 2009 p. 566–91.

[18] Nelissen MHJ. Structure of the dominance hierarchy and dominance determining group factors in *Melanochromic auratus* (Pices, Cichlidae). Behav. 1985;94:85–107. 10.1163/156853985x00280

[19] Oliveira RF, Almada VC. On the (in)stability of dominance hierarchies in the cichlid fish *Oreochromis mossambicus*. Agressive Behav. 1996;22:37–45. 10.1002/(SICI)1098-2337(1996)22:1<37::AID-AB4>3.0.CO;2-R

[20] Chase ID. Behavioral sequences during dominance hierarchy formation in chickens. Science. 1982a;216:439–40. 10.1126/science.216.4544.439 17745870

[21] Bonabeau E, Theraulaz G, Deneubourg J-L. Mathematical model of self-organizing hierarchies in animal societies. Bull Math Biol. 1996;58:661–717. 10.1016/0092-8240(95)00364-9

[22] Bonabeau E, Theraulaz G, Deneubourg J-L. Dominance orders in animal societies: The self-organization hypothesis revisited. Bull Math Biol. 1999;61:727–57. 10.1006/bulm.1999.0108 17883222

[23] Dugatkin LA. Winner and loser effects and the structure of dominance hierarchies. Behav Ecol. 1997;8:583–7. 10.1093/beheco/8.6.583

[24] Hemelrijk CK. An individual-orientated model of the emergence of despotic and egalitarian societies. Proc R Soc Lond, Ser B: Biol Sci. 1999;266:361–9. 10.1098/rspb.1999.0646

[25] Hemelrijk CK. Towards the integration of social dominance and spatial structure. Anim Behav. 2000;59:1035–48. 10.1006/anbe.2000.1400 10860531

[26] Skvoretz J, Faust K, Fararo TJ. Social, structure, networks, and E-state structuralism models. J Math Soc. 1996;21:57–76. 10.1080/0022250x.1996.9990174

[27] Broom M, Cannings C. Modelling dominance hierarchy formation as a multi-player game. J Theor Biol. 2002;219(3):397–413. 10.1111/j.1469-1809.1999.ahg634_0351_4.x 12419665

[28] Chase ID, Tovey C, Murch P. Two's company, three's a crowd: Differences in dominance relationships in isolated versus socially embedded pairs of fish. Behav. 2003;140(10):1193–217. 10.1163/156853903771980558

[29] Johnsson JI. Individual recognition affects aggression and dominance relations in rainbow trout, *Oncorhynchus mykiss*. Ethology. 1997;103(4):267–82.

[30] Miklósi Á, Haller J, Csányi V. Learning about the opponent during aggressive encounters in paradise fish (*Macropodus opercularis* L.): When it takes place? Behav Process. 1997;40(1):97–105. 10.1016/S0376-6357(96)00755-3 24897617

[31] Landau HG. On dominance relations and structures of animal societies. Bull Math Biophys. 1951;13:1–19. 10.1007/bf02478336

[32] Chase ID. Models of hierarchy formation in animal societies. Behav Sci. 1974;19(6):374–82. 10.1002/bs.3830190604

[33] Ang TZ, Manica A. Unavoidable limits on group size in a body size-based linear hierarchy. Behav Ecol. 2010;21:819–25. 10.1093/beheco/arq062

[34] Lindquist WB, Chase ID. Data-based analysis of winner-loser models of hierarchy formation in animals. Bull Math Bio. 2009;71(13):556–84. 10.1007/s11538-008-9371-9 19205807

[35] Milo R, Shenn-Orr S, Itzkowitz S, Kashtan N, Chklovskii D, Alon U. Network motifs: Simple building blocks of complex networks. Science. 2002;298:824–7. 10.1126/science.298.5594.824 12399590

[36] Alon U. Network motifs: Theory and experimental approaches. Nat Rev Genet. 2007;8:450–61. 10.1038/nrg2102 17510665

[37] Johnsen EC. The micro-macro connection: Exact structure and process. Ima V Math. 1989;17:169–201. 10.1007/978-1-4684-6381-1_7

[38] Martin JL. Social structures. Princeton, NJ: Princeton University Press; 2009.

[39] Chartrand G. Introductory graph theory. New York: Dover; 1984.

[40] Wilson EO. Sociobiology. Cambridge, MA: Harvard University Press; 1975.

[41] McDonald DB, Shizuka D. Comparative transitive and temporal orderliness in dominance networks. Behav Ecol. 2013;24:511–20. 10.1093/beheco/ars192

[42] Chase ID. The sequential-analysis of aggressive acts during hierarchy formation—an application of the jigsaw puzzle approach. Anim Behav. 1985;33:86–100. 10.1016/S0003-3472(85)80122-6

[43] Bradbury JW, Vehrencamp SL. Complexity and behavioral ecology. Behav Ecol. 2014;25(3):435–42. 10.1093/beheco/aru014

[44] Goldenfeld N, Kadanoff LP. Simple lessons from complexity. Science. 1999;284(5411):87–9. 10.1126/science.284.5411.87 10102823

[45] Ulanowicz RE. A third window. West Conshohocken, PA: Templeton Foundation Press; 2009.

[46] Anderson PW. More is different. Science. 1972;177(4047):393–6. 10.1126/science.177.4047.393 17796623

[47] Chase ID, Rohwer S. Two methods for quantifying the development of dominance hierarchies in large groups with applications to Harris sparrows. Anim Behav. 1987;35(4):1113–28. 10.1016/s0003-3472(87)80168-9

[48] So N, Franks B, Lim S, Curley JP. A social network approach reveals associations between mouse social dominance and brain gene expression. PLoS One. 2015;10(7): e0134509 10.1371/journal.pone.0134509 26226265 PMC4520683

[49] Grosenick L, Clement TS, Fernald RD. Fish can infer social rank by observation alone. Nature. 2007;445:429–32. 10.1038/nature05646 17251980

[50] Oliveira RF, McGregor PK, Latruffe C. Know thine enemy: Fighting fish gather information from observing conspecific interactions. Proc Royal Soc London, Series B—Bio Sci. 1998;265(1401):1045–9. 10.1098/rspb.1998.0397

[51] Paz-y-Mino G, Bond AB, Kamil AC, Balda RP. Pinyon jays use transitive inference to predict social dominance. Nature. 2004;430(7001):778–81. 10.1038/nature02723 15306809

[52] Chase ID, Weissburg M, DeWitt TH. The vacancy chain process: A new mechanism of resource allocation in animals with application to hermit crabs. Anim Behav 1988;36:1265–74. 10.1016/S0003-3472(88)80195-7

[53] Lewis SM, Rotjan RD. Vacancy chains provide aggregate benefits to Coenobita clypeatus hermit crabs. Ethology. 2009;115(4):356–3365. 10.1111/j.1439-0310.2009.01626.x

[54] Briffa M. The influence of personality on a group-level process: Shy hermit crabs make longer vacancy chains. Ethology. 2013;119(11):1014–23. 10.1111/eth.12148

[55] Chase ID. Vacancy chains. Annu Rev Sociol. 1991;17:133–54. 10.1146/annurev.so.17.080191.001025

